# A Vaccine of L2 Epitope Repeats Fused with a Modified IgG1 Fc Induced Cross-Neutralizing Antibodies and Protective Immunity against Divergent Human Papillomavirus Types

**DOI:** 10.1371/journal.pone.0095448

**Published:** 2014-05-06

**Authors:** Xue Chen, Hongyang Liu, Ting Zhang, Yanchun Liu, Xixiu Xie, Zhirong Wang, Xuemei Xu

**Affiliations:** Department of Biophysics and Structural Biology, Institute of Basic Medical Sciences Chinese Academy of Medical Sciences, School of Basic Medicine Peking Union Medical College, Beijing, China; International Centre for Genetic Engineering and Biotechnology, Italy

## Abstract

Current human papillomavirus (HPV) major capsid protein L1 virus-like particles (VLPs)-based vaccines in clinic induce strong HPV type-specific neutralizing antibody responses. To develop pan-HPV vaccines, here, we show that the fusion protein E3R4 consisting of three repeats of HPV16 L2 aa 17–36 epitope (E3) and a modified human IgG1 Fc scaffold (R4) induces cross-neutralizing antibodies and protective immunity against divergent HPV types. E3R4 was expressed as a secreted protein in baculovirus expression system and could be simply purified by one step Protein A affinity chromatography with the purity above 90%. Vaccination of E3R4 formulated with Freunds adjuvant not only induced cross-neutralizing antibodies against HPV pseudovirus types 16, 18, 45, 52, 58, 6, 11 and 5 in mice, but also protected mice against vaginal challenges with HPV pseudovirus types 16, 45, 52, 58, 11 and 5 for at least eleven months after the first immunization. Moreover, vaccination of E3R4 formulated with FDA approved adjuvant alum plus monophosphoryl lipid A also induced cross-neutralizing antibodies against HPV types 16, 18 and 6 in rabbits. Thus, our results demonstrate that delivery of L2 antigen as a modified Fc-fusion protein may facilitate pan-HPV vaccine development.

## Introduction

More than 150 HPV genotypes have been identified with different epithelial tropisms [1]. Infection with cutaneous HPV types (such as HPV1, 2, 3, 5) causes benign cutaneous warts or epidermodysplasia verruciformis (HPV5). Mucosal HPV types infect the upper part of the respiratory tract (HPV6, 11), oral cavity (HPV13), and the epithelium of the anogenital region. The low-risk anogenital HPVs (such as HPV6, 11, 42) cause genital warts whereas the high-risk HPVs (such as HPV16, 18, 31, 45, 52, 58) are associated with progression to carcinoma of the cervix, vulva, vagina, penis, anus and oropharynx [2]. Cervical cancer is the third most common cancer worldwide, and about 70% of cervical cancers are caused by infections with HPV types 16 and 18.

Currently there are two licensed HPV major capsid protein L1 virus-like particle (VLP)-based vaccines, Cervarix, a bivalent HPV16/18 vaccine, and Gardasil, a quadrivalent HPV16/18/6/11 vaccine. Although some cross-reactivity has been observed between closely related HPV genotypes, the protection provided upon vaccination with HPV L1 VLP vaccine is largely HPV type-specific, indicating that vaccination provides very little cross-protection to the HPV types not covered by the vaccines [3], [4]. The limited cross-protective capacity of L1-based vaccines makes it necessary to develop a pan-HPV vaccine.

Vaccination with recombinant minor capsid protein, L2, or peptides derived from L2 results in the production of cross-neutralizing antibodies that are protective in animal models [5], [6]. In the context on native virions, L2 is poorly immunogenic. Neither natural infection nor immunization with HPV L1/L2 VLPs elicits anti-L2 antibody responses [7]. Studies showed that L2 is poorly exposed on the surface of virions. It is generally accepted that after HPV virus binds to heparin sulfate moieties on the basement membrane, the capsid undergoes a conformational change that exposes the amino terminus of L2 [8]. The exposed N-terminus of L2 is susceptible to protease cleavage, thus exposing L2 epitopes near the N-terminus of the protein [9]. Several regions in the N-terminus of L2 can be targeted by neutralizing antibodies [10], [11], [12], [13], [14], which prevent viruses from transferring from basement membrane to unidentified receptor on epithelial cells. A major cross-neutralizing epitope located in amino acid 17 to 36 represents an attractive candidate antigen for broadly protective vaccination [15].

The neutralizing titers produced by L2 vaccination are considerably lower than that induced by L1 VLP vaccination, particularly against heterologous HPV types [16]. Therefore, it is likely that an L2 vaccine will only be effective if its immunogenicity is enhanced. B-cell activation is initiated following engagement of the B-cell receptor (BCR) by a specific antigen. Large antigens, such as immune complexes and viruses, can be presented to B cells more efficiently than small soluble molecules [17], [18], [19]. Unlike T-cell receptor (TCR) which recognizes processed epitopes in the context of major histocompatibility complex molecules, BCR may recognize unprocessed antigens presented on the surface of antigen presenting cells (APCs) [20], [21], [22]. Displaying multivalent L2 epitope in exposed regions on VLPs derived from papillomavirus [23], [24], [25], bacteriophage [26], [27] and adeno-associated virus [28], or in the surface region of bacterial thioredoxin [10] has shown to induce enhanced epitope-directed antibody responses and broadly protective immunity.

**Figure 1:**
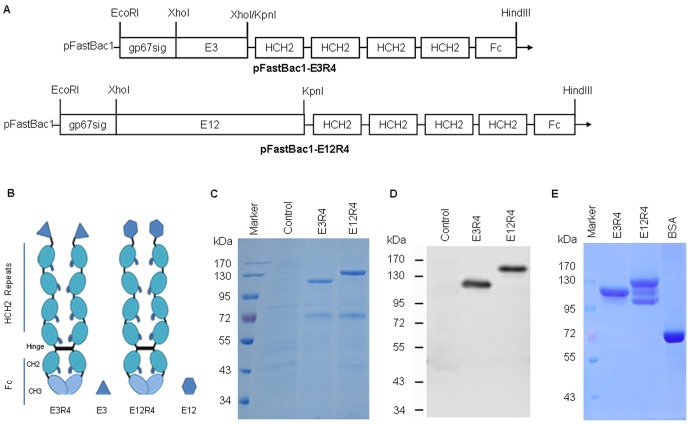
Structure and expression of L2R4 proteins. Schematic diagram of pFastBac1-E3R4 and pFastBac1-E12R4 constructs (each L2R4 gene is preceded by a signal peptide sequence from baculovirus gp67 for protein secretion) (A). E3R4 or E12R4 consists of three or twelve repeats of HPV16 L2 aa 17–36 and a modified human IgG1 Fc (R4). The R4 is a tandem repeat composed of 4 copies of the hinge and CH2 sequences (HCH2) from IgG fused to an Fc region of IgG (B). The expression of E3R4 and E12R4 in culture medium were performed by reducing SDS-PAGE with Coomassie blue staining (C), and Western blot (D) using RG-1 (1∶5,000). The recombinant proteins were purified by rProtein A affinity column, and the purity was examined by SDS-PAGE with Coomassie blue staining. 10 µg of each L2R4 protein or 12 µg of BSA was loaded (E). Control, Sf9 culture medium; BSA, bovine serum albumin.

The Fc receptors for IgG (FcγRs), expressed on dendritic cells (DCs) and APCs, can bind and internalize antigen-IgG immune complexes via the interaction with the IgG, resultsing in enrichment of exogenous antigens in DCs, which facilitates DC maturation and antigen-specific T cell responses and humoral responses. Recombinant antigen-immunoglobin Fc-fusion proteins were shown to increase the immunogenicity of the fused antigens and elicit neutralizing antibody responses to HIV [29], [30] and protective immunity to virulent herpes simplex virus [31], influenza viruses [32] and Ebola viruses [33]. In this study, we showed for the first time that fusing HPV16 L2 aa 17–36 epitope repeats to a recombinant ligand for FcγRs (designated L2R4, see Figure 1A–B) could significantly increase the immunogenicity of the L2 peptide and induce cross-neutralizing antibodies and protective immunity against a range of phylogenetically distant HPV types.

## Materials and Methods

### Antigen preparation

A recombinant ligand for FcγRs, R4, is an Fc-like fusion protein that contains a tandem repeat composed of 4 copies of the hinge and CH2 regions (HCH2) of human IgG1 for each chain [34], and the HCH2 region of IgG encompasses the sequences that bind FcγRs [34], [35], [36], [37]. To express L2 peptide as an Fc-like fusion protein, the L2 antigen gene encoding three repeats or twelve repeats of HPV16 L2 aa 17–36 epitope with a three amino acid linker (Gly-Gly-Pro) in between was fused in-frame to the upstream of R4 (Figure 1A), and the resulting fusion protein was designated as E3R4 or E12R4 (Figure 1B). To facilitate purification, the signal peptide sequence of gp67 of baculovirus was added to the upstream of each L2R4 fusion gene. The L2R4 genes were codon optimized based on the codon usage bias in Sf9 cells (Sangon, Shanghai), subcloned into pFastBac1 vector and expressed as secreted proteins in baculovirus expression system as described previously [7], [38], [39]. E3R4 and E12R4 proteins were purified from the culture supernatant by rProtein A sepharose affinity chromatography according to the manufacturer's instructions (GE Healthcare). R4 protein was also expressed and purified as a scaffold control. The expression and analysis of the recombinant proteins were evaluated by SDS-PAGE with Coomassie blue staining and Western blot with 1∶5,000 dilution of RG-1, a cross-neutralizing and protective monoclonal antibody that recognizes residues 17-36 of HPV16 L2 (generously provided by Richard Roden) [15] as previous reported [38]. The purity of purified proteins was determined by Sf9 host cell protein (HCP) ELISA as described previously [39].

### Animal immunizations

Four- to six-week-old female BALB/c mice and New Zealand white rabbits were purchased from the Institute of Laboratory Animal Science, Chinese Academy of Medical Sciences, and kept in the animal facility of the Institute of Basic Medical Sciences, Chinese Academy of Medical Sciences. All animal work was done in accordance with the guidelines of the Institutional Animal Care and Use Committee of the Institute of Laboratory Animal Science, Chinese Academy of Medical Sciences, and all experimental protocols were approved by the Institutional Animal Care and Use Committee.

Groups of 5 mice were vaccinated on weeks 0, 2, 4 and 6 with 1.7 nmol of E3R4, E12R4, R4, E3 peptide (Lifetin, Beijing) or phosphate-buffered saline (PBS) formulated with complete Freunds adjuvant (CFA) for priming dose and with incomplete Freunds adjuvant (IFA) for booster immunizations. Groups of 2 rabbits were vaccinated subcutaneously on weeks 0, 2, 4 and 6 with 4 nmol of E3R4 alone or with Alum (Sigma-Aldrich) and monophosphoryl lipid A (Sigma-Aldrich) (Alum-MPL) adjuvant. Sera were collected at weeks 6 and 8 and stored at -20°C.

### HPV pseudovirus (PsV) preparation

Pseudoviruses of HPV16, 18, 45, 58, 6, 11 and 5 (PsV16, PsV18, PsV45, PsV58, PsV6, PsV11 and PsV5, respectively) with encapsidated reporter plasmid pLucf which encoding both luciferase and green fluorescence protein (GFP), or plasmid pEGFP-N1 (Clonetech) which encoding GFP were produced in 293TT cells as previously described [38], [40], [41] with minor modifications. For PsV52 preparation, 16 µg DNA (8 µg of shell plasmid and 8 µg of reporter plasmid) was mixed with 56 µl of TurboFect (Thermo Fisher) and 1.6 ml of unsupplemented DMEM, then added to 6.5×10^6^ 293TT cells as suggested by Prof. Reinhard Kirnbauer. The titer of PsV (infectious units per ml, IU/ml) was determined by GFP expression in 293TT cells as described in http://home.ccr.cancer.gov/lco/pseudovirusproduction.htm. L1/L2 expression plasmids, pLucf plasmid, were generously provided by John Schiller, Susana Pang, Chris Buck, Martin Müller and Tadahito Kanda.

### Enzyme-Linked Immunosorbent Assays (ELISA)

To assess the binding of L2R4 proteins to RG-1, ELISA plates were coated with 100 ng of each protein (E3R4, E12R4, R4), or 5 ng (equivalent to the amount of the recombinant proteins on a mole basis) of E3 peptide at 4°C overnight. ELISA was performed as previously described [38], [39]. Briefly, wells were blocked with 5% bovine serum albumen (BSA) in PBST at room temperature for 2 hours. RG-1 monoclonal antibody (1∶3,000 dilution) was added to each well and incubated at room temperature for 2 hours. After wash, wells were incubated with horseradish peroxidase (HRP)-conjugated goat anti-mouse IgG (1∶5,000 dilution) at room temperature for 1 hour. Plates were developed by the addition of 0.4 g/ml of O-phenylenediamine diluted in phosphate-citrate buffer, pH 5.0, containing 0.045% (v/v) hydrogen peroxide. The enzymatic reaction was stopped by the addition of 50 µl of a solution containing 2 M H_2_SO4. Plates were read at 490 nm, and antibody titer was determined as the reciprocal of the highest serum dilution with an OD 490 greater than 0.2 and 2-fold higher than control sera at the same dilution.

To measure cross-reactive antibody responses to HPV capsids in antisera, ELISA plates were coated with 1.5×10^4^ to 2.0×10^4^ IU of HPV PsVs (depending on the PsV stock) encoding GFP. Sera collected at week 8 were assessed by end-point dilution ELISA as described above. Binding titer was determined as the reciprocal of the highest serum dilution with an OD 490 greater than 0.2 and 2-fold higher than control sera at the same dilution. Statistical significance was determined by one-way ANOVA and Bonferroni multiple comparison test (GraphPad Prism 5). *P* value <0.05 was considered to be statistically significant.

### PsV-based neutralization assays

HPV PsV-based neutralization assays were performed as previous reports [38], [39], [41]. Pseudovirus (encoding GFP) diluents and serially diluted sera were mixed and added to the cell culture plate, after incubation, the 293TT cells were digested with trypsin and transferred to cell sorting tube. The fluorescent cells were detected by fluorescence activated cell sorting. The endpoint titer was calculated as the reciprocal of the highest serum dilution with percent infection inhibition higher than 50%, and a titer <50 was considered as nonsignificant [42], [43]. Every sample was detected in duplicate. Statistical significance was determined by one-way ANOVA and Bonferroni multiple comparison test.

### Murine vaginal HPV PsV challenge

Mice were treated with 3 mg of progesterone subcutaneously four days before PsV challenge. The immunized mice were intravaginally pretreated with 50 µl of 4% nonoxynol-9 (N9, Igepal, Sigma) at six hours prior to PsV challenge, R4 and PBS injected female mice were used as controls. 20 µl of PsV preparation containing 2.5×10^5^ to 1.0×10^7^ IU of PsVs (encapsidated reporter plasmid pLucf) and 1% carboxymethyl cellulose (CMC, Sigma) was intravaginally instilled using a positive-displacement pipette. Forty-eight hours post-PsV challenge, mice were vaginally instilled with 0.4 mg of 5′-F-Luciferin (CellCyto Life Sciences). Three minutes later, luciferase signals were acquired for 5 min with a biofluorescence imaging (BFI) system of the LB 983 NightOWL II (Berthold Technologies), and analyzed with IndiGO 2 software (Berthold Technologies). Statistical significance was determined by one-tailed unpaired t-test.

## Results

### Preparation of L2R4 proteins

We first examined the expression of L2R4 proteins in the culture medium by SDS-PAGE since they are expressed as secreted proteins. We observed E3R4 (120 kDa) or E12R4 (130 kDa) band by Coomassie blue staining (Figure 1C), which was further confirmed by Western blot (Figure 1D). The recombinant proteins were highly expressed, with approximately 3 mg of E3R4 or 2.5 mg of E12R4 obtained per 100 ml culture medium. The proteins were purified with rProtein A affinity chromatography, and the purity was analyzed by SDS-PAGE with Coomassie blue staining (Figure 1E) and Sf9 HCP ELISA [39]. When 10 µg of each protein was loaded, a single band was observed in E3R4 sample, indicating that E3R4 was stable, but two additional small bands were also observed in E12R4 sample, indicating that E12R4 was partially degraded as these small bands were all reactive with RG-1 antibody by Western blot (not shown). By HCP ELISA, we found that the purity of purified E3R4 and E12R4 was above 90%. To further characterize the stability of E3R4, we kept the purified protein at room temperature for 5 days and found a single band in E3R4 sample by SDS-PAGE with Coomassie blue staining (not shown). Taken together, we conclude that E3R4 fusion protein can be highly expressed and simply purified with stability *in vitro*.

### L2R4 proteins induced cross-reactive antibodies against divergent HPV types

**Figure 2:**
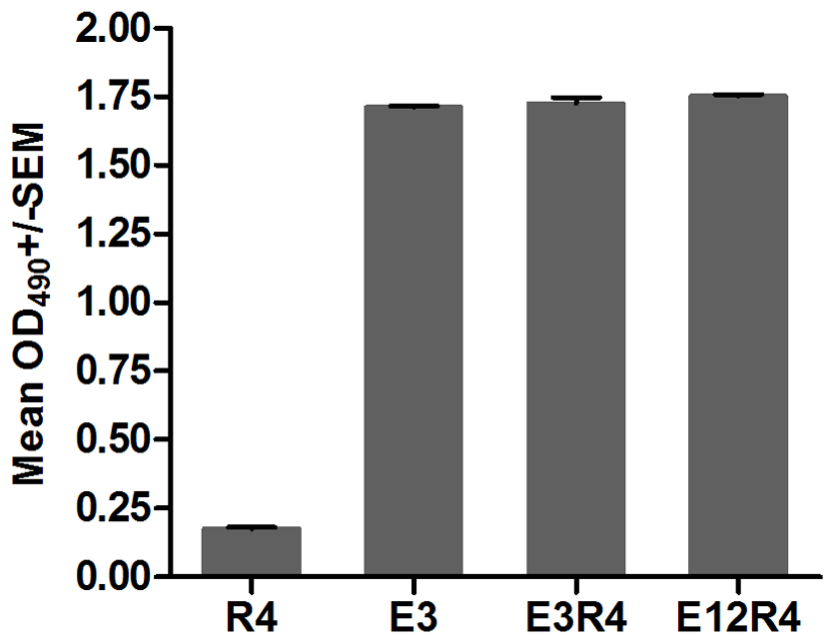
Reactivity of RG-1 with L2R4 proteins. ELISA plates were coated with 100(E3R4, E12R4, R4), or 5 ng of E3 peptide (equivalent to the amount of R4 based proteins on a mole basis). Binding of a 1∶3,000 dilution of RG-1 was detected with HRP-conjugated goat-anti-mouse IgG. Reactivity was determined by measuring the mean optical density (OD) values at 490 nm. Error bars indicate standard error of the mean (SEM) of triplicate wells. Results are expressed as mean±SD. The experiment was repeated twice.

**Figure 3:**
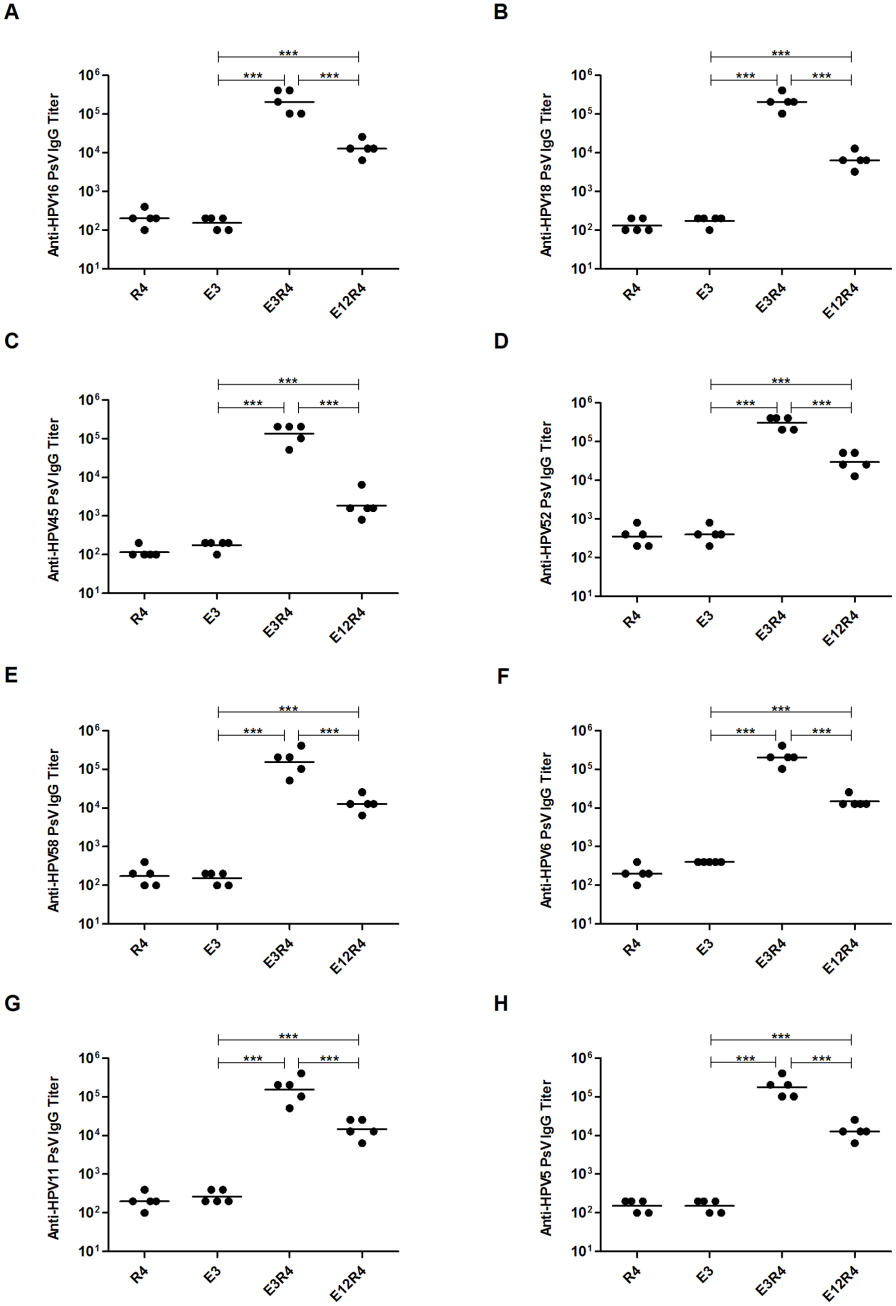
Cross-reactive antibody titers in sera from mice vaccinated with L2R4 proteins. Mice were immunized on weeks 0, 2, 4 and 6 with each protein (E3R4, E12R4 and R4) or E3 peptide formulated with Freunds adjuvant, and sera were collected at week 8. Dilutions of each of HPV L1/L2 pseudovirus, including PsV16, 18, 45, 52, 58, 6, 11 and 5, were coated in ELISA plates and used to determine the cross-reactive antibody titers of the antiserum. The binding titer was determined as the reciprocal of the highest serum dilution with an OD 490 greater than 0.2 and 2-fold higher than control sera at the same dilution. The experiment was repeated twice. The horizontal bars represent the geometric mean antibody titers. The statistically significant differences (using one-way ANOVA and Bonferroni multiple comparison test) were indicated by: ***, *P*<0.001.

To assess the immunogenicity of L2R4 proteins, we first showed that RG-1 strongly bound to E3R4, E12R4 and E3 peptide, but not to R4 scaffold control (Figure 2), indicating that L2 epitopes in L2R4 proteins are well exposed to be recognized by RG-1 antibody. We next asked whether L2R4 proteins were able to induce cross-reactive antibody responses. As our goal was to develop a vaccine inducing the broadest cross-reactivity against divergent HPV types, a panel of HPV PsVs, including HPV16, 18, 45, 52, 58 (five common oncogenic types), HPV6, 11 (the most common types found in benign genital warts) and HPV5 (causes epidermodysplasia verruciformis) were used to coat ELISA plates. Sera were collected at 2 weeks after four immunizations from mice, and the HPV capsid-reactive antibody titer was measured by endpoint dilution ELISA. The binding titers against each PsV type in E3R4 and E12R4 antisera were significantly higher than E3 antisera (*P*<0.001) (Figure 3), suggesting that the R4 scaffold can enhance the immunogenicity of fused antigens. We also observed stronger antibody responses induced by E3R4 than E12R4 (*P*<0.001), which may be due to the instability of E12R4 (Figure 1E). Thus, these data show that E3R4 fusion protein is highly immunogenic and induces broad cross-reactive antibodies against divergent HPV types.

### E3R4 induced cross-neutralizing antibody responses in mice

**Figure 4:**
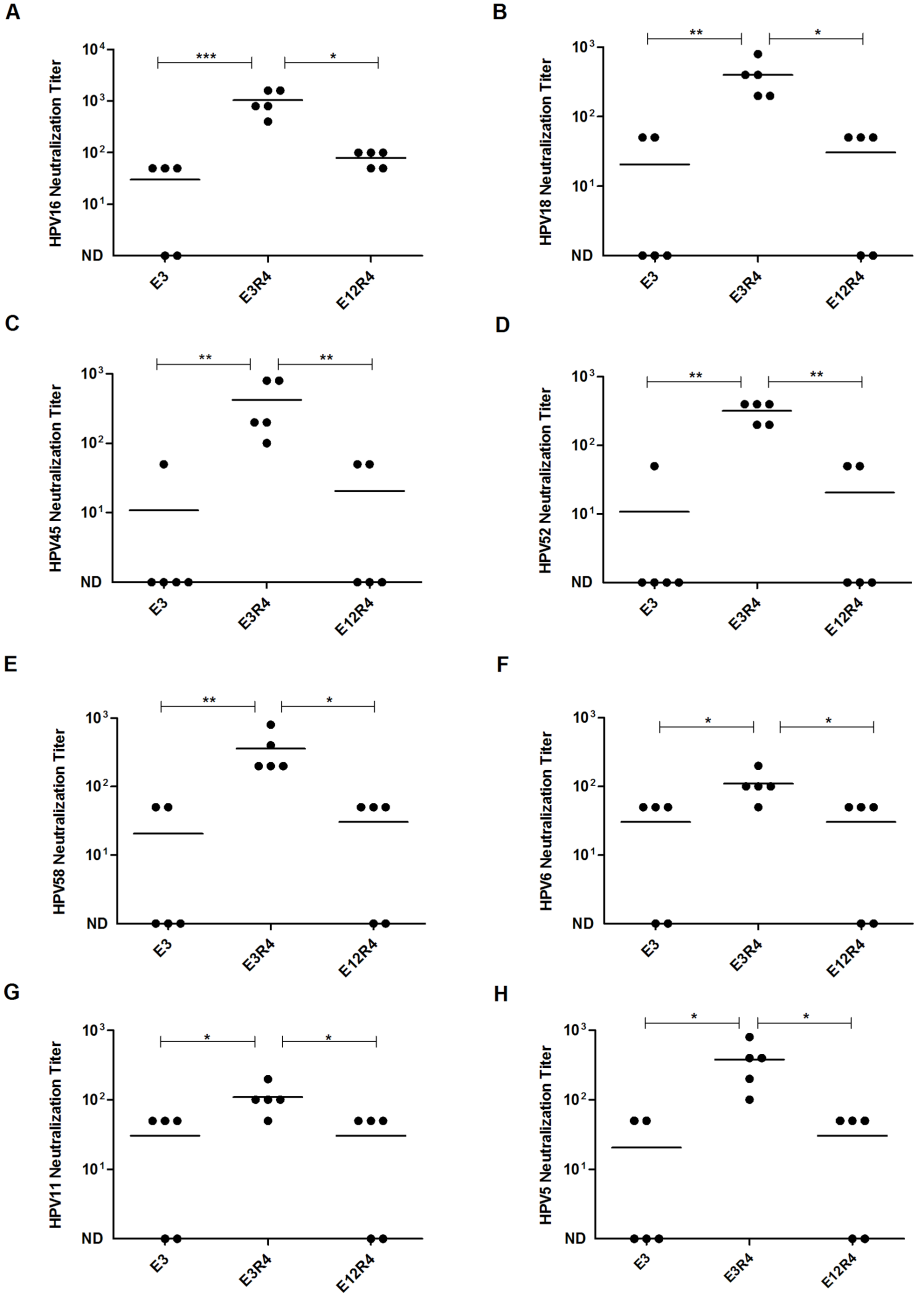
Neutralizing antibody titers in sera of mice vaccinated with L2R4 proteins. Mice were immunized and sera were collected (see Figure 3 legend for detail). Antisera were tested for in vitro neutralization titers against HPV16 (A), HPV18 (B), HPV45 (C), HPV52 (D), HPV58 (E), HPV6 (F), HPV11 (G) and HPV5 (H) pseudovirus. The horizontal bars represent the geometric mean antibody titers. The statistically significant differences (using one-way ANOVA and Bonferroni multiple comparison test) were indicated by: *, *P*<0.05; **, *P*<0.01; ***, *P*<0.001. ND, not detectable.

HPV capsid-based ELISA assay detects both non-neutralizing and neutralizing antibodies, and the neutralizing antibodies are thought to be the primary immune mechanism of protection by HPV vaccination. Thus, we further examined the cross-neutralizing antibody responses induced by L2R4 proteins using PsV-based *in vitro* neutralization assay. As proof-of-concept, a panel of PsVs encoding GFP from divergent HPVs, including HPV16, 52, 58 (alpha-9), HPV6, 11 (alpha-10), HPV18, 45 (alpha-7) and HPV5 (beta-1), were used in neutralization assays. Although the cross-neutralizing antibody levels in the antisera after the third immunization of E3R4 or E12R4 were very low (titer, <50, not shown), we did observed broad cross-neutralizing antibody responses induced by E3R4 after the fourth immunization (Figure 4). The mean neutralizing antibody titers of the E3R4 antisera against HPV16, 52, 58, 6, 11, 18, 45 and 5 were 1040, 320, 360, 110, 110, 400, 420 and 380, respectively, which were significantly higher than E12R4 or E3 antisera (*P*<0.05). E12R4 only induced weak anti-HPV16 neutralizing antibody response (mean titer, 80) and the neutralizing antibody titers against heterologous PsVs in E12R4 antisera were all below 50. Although E12R4 elicited significantly higher cross-reactive antibody responses than E3 measured by HPV capsid-based ELISA assay (Figure 3), there were no significant differences between the cross-neutralizing antibody titers in E12R4 and E3 antisera (*P*>0.05), suggesting that the majority of serum antibodies induced by E12R4 were not neutralizing antibodies. We didn't detect any neutralizing antibodies in R4 scaffold control antisera (not shown). Collectively, we show that Freunds adjuvanted E3R4 vaccine induces broad cross-neutralizing antibody responses in mice.

### E3R4 immunization protected mice from HPV PsVs challenges

**Figure 5:**
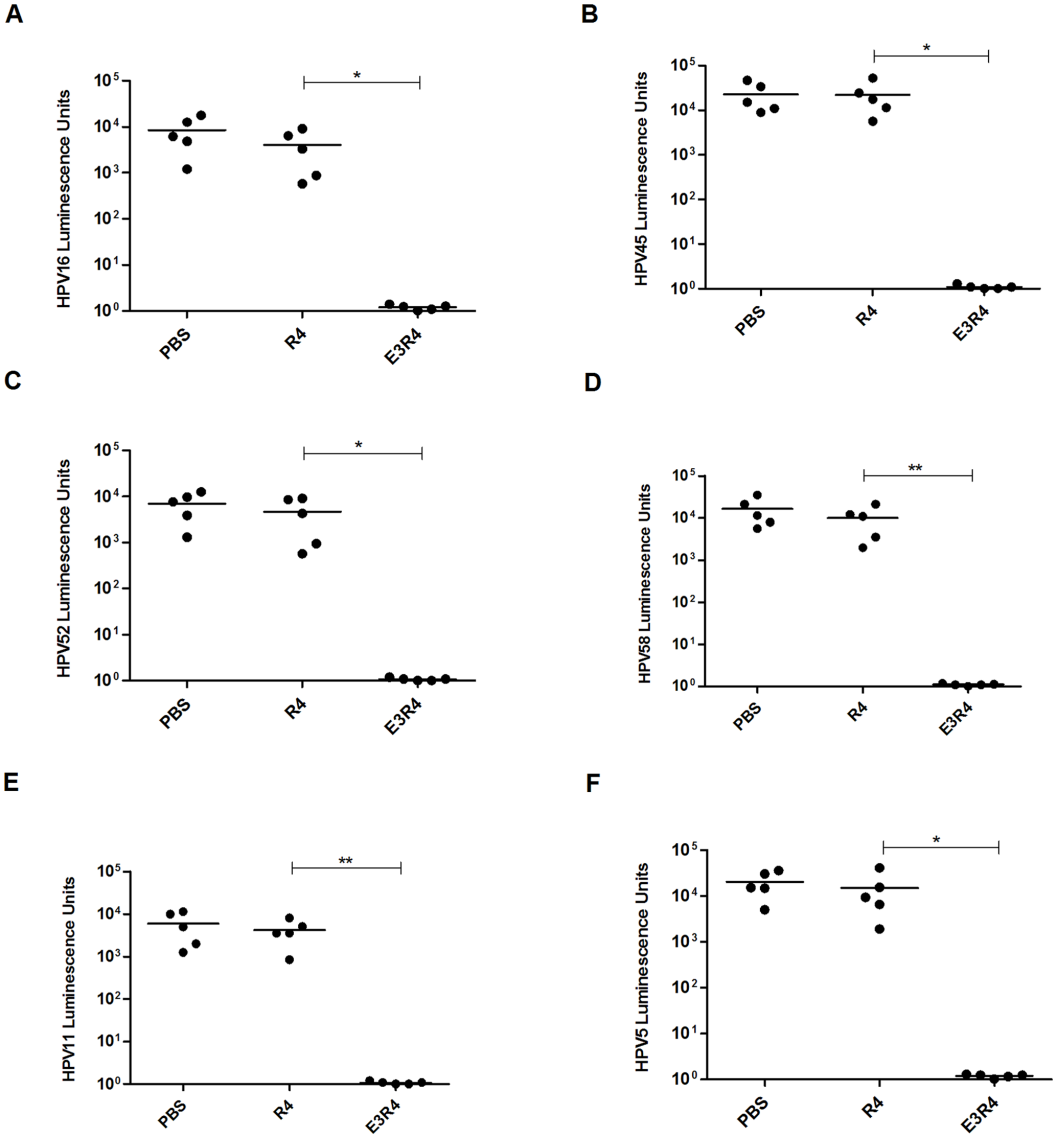
Broad-spectrum protection against in vivo genital HPV PsV challenges. Mice were immunized four times with E3R4, R4 or phosphate-buffered saline (PBS) with CFA/IFA adjuvant. At eleven months after the first immunization, mice were intravaginally challenged with HPV16 (A), HPV45 (B), HPV52 (C), HPV58 (D), HPV11 (E), or HPV5 (F) pseudovirus. Luciferase signals were acquired for 5 min with a LB 983 NightOWL II imager 48 h after PsV infection. Infection is measured as bioluminescence. The statistically significant differences (using one-tailed unpaired t-test) were indicated by: *, *P*<0.05; **, *P*<0.01.

Given the broad cross-neutralizing antibody responses elicited by Freunds adjuvanted E3R4 protein in mice, we next examined the *in vivo* protection against multiple HPV types using the vaginal challenge assay. Mice were immunized with E3R4 or R4 formulated with Freunds adjuvant for four times with 2-week intervals. Eleven months after the first immunization, mice were vaginally challenged with divergent PsVs (HPV16, 45, 52, 58, 11, 5) encoding luciferase. Consistent to relatively high-levels of cross-neutralizing antibodies in antisera (Figure 4), mice immunized with E3R4 were completely protected from challenge of PsV16 (Figure 5A, *P*<0.05), PsV45 (Figure 5B, *P*<0.05), PsV52 (Figure 5C, *P*<0.05), PsV58 (Figure 5D, *P*<0.01), PsV11 (Figure 5E, *P*<0.01) and PsV5 (Figure 5F, *P*<0.05), whereas the mean luminescence signal in mice immunized with R4 scaffold was comparable to that in PBS control mice. Thus, our data demonstrate that E3R4 vaccine could provide substantial protection against diverse types of HPV PsVs.

### E3R4 induced cross-neutralizing antibody responses in rabbits

To further evaluate the potency and potential clinical application of E3R4 fusion protein, we examined the immune responses induced by E3R4 formulated with FDA approved adjuvant Alum-MPL in another animal model, the New Zealand white rabbit. Two rabbits were vaccinated with either E3R4 alone or formulated with Alum-MPL according to previous studies [24], [44]. Sera collected after the fourth immunization were analyzed by PsV-based *in vitro* neutralization assay. The results were shown in Table 1. While the neutralizing antibody titers against PsV16 and other PsV types were all below 25 in E3R4 alone antisera, we observed neutralizing antibody responses against HPV16, 18 and 6 in antisera from rabbits immunized with E3R4 formulated with Alum-MPL. The neutralizing antibody titers of the two rabbits were 400 and 50 against HPV16, 50 and 25 against HPV18, 50 and less than 25 against HPV6. The neutralizing antibody titers against other PsV types were all below 25. We did notice that the cross-neutralizing antibody responses by E3R4 in rabbits were weaker than in mice, which may be due to the fact that Alum-MPL adjuvant used in rabbits is less effective than Freunds adjuvant used in mice. In addition, reported antigen doses used in rabbits were usually 5-fold-higher or more than in mice [24], [43], [45], but the E3R4 dose in rabbits is only 2.35-fold-higher than that in mice. Thus, the relatively lower dose injected in rabbits may also cause the weaker cross-neutralizing antibody responses than in mice. Nevertheless, our accumulative data show that E3R4 induces cross-neutralizing antibodies in two different animal models when formulated with Alum-MPL or Freunds adjuvants.

**Table 1:** Neutralizing antibody titers in sera of rabbits vaccinated with E3R4 alone or with Alum-MPL adjuvant.

Pseudovirions	Neutralizing antibody titer			
	E3R4 with Alum-MPL		E3R4 alone	
	1	2	1	2
HPV16	400	50	<25	<25
HPV52	<25	<25	<25	<25
HPV58	<25	<25	<25	<25
HPV18	50	25	<25	<25
HPV45	<25	<25	<25	<25
HPV6	50	<25	<25	<25
HPV11	<25	<25	<25	<25
HPV5	<25	<25	<25	<25

Groups of 2 rabbits were immunized four times with E3R4 alone or with Alum-MPL adjuvant, and sera were collected at two weeks after the fourth immunization. Antisera of two rabbits (nos. 1 and 2) were tested for cross-neutralization of eight HPV pseudovirus types. Neutralization titers were determined as previously described [38], [39], [41].

Abbreviations: Alum-MPL, Alum and monophosphoryl lipid A; HPV, human papillomavirus.

## Discussion

Neutralizing antibodies binding to linear epitopes in HPV16 L2 aa 17–36, 65–81 and 108–120 have been described [11], [15], [23], [42]. Passive transfer of HPV16 L2 aa 17–36 antiserum or RG-1 antibody has shown to protect the mice from HPV16 PsV infection, suggesting that the neutralizing antibodies induced by L2 epitope is sufficient for *in vivo* protection [15], [42]. It was reported that sera from mice immunized with a tandem repeat of L2 aa 17–36 derived from 22 different HPV types with GPI-0100 adjuvant could neutralize HPV PsV types 16, 18, 45, but not types 6, 58 [43]. In this study, we fused three repeats of HPV16 L2 aa 17–36 with a modified IgG1 Fc to generate E3R4 fusion protein, which can be highly expressed, simply purified with high purity and is relatively stable *in vitro*. Importantly, immunization of E3R4 with Freunds adjuvant elicited broad neutralizing antibody responses against HPV types 16, 18, 45, 52, 58, 6, 11 and 5 in mice. Induction of long-term and broadly protective immunity against all clinically relevant HPV types is the goal of pan HPV vaccine development. Similar to the RG1-VLPs (presenting RG-1 epitope on the surface of HPV16 L1 VLPs) which were shown to induce enduring protective antibody against HPV58 for 12 month after vaccination [25], our results also showed that the broadly protective immunity induced by E3R4 with Freunds adjuvant sustained at least 11 months after the first immunization, highlighting that L2 epitope-based protein vaccine is able to induce long-lasting protective immunity when the epitope is properly delivered.

In the current study, we used R4, a modified IgG1 Fc, as a scaffold to display L2 epitopes and observed strong effects of R4 scaffold on enhancing the immune responses induced by L2 epitopes. Fc of IgG is considered as an important fusion tag for co-expressing several viral proteins to promote correct folding of the fusion proteins, facilitate purification, enhance the binding to APCs expressing FcγR and improve the immunogenicity of fused proteins [30], [32], [33], [46], [47]. Previous studies showed that recombinant proteins by fusing R4 to antigen proteins, such as human serum albumin domain 1 and the clostridal botulinum neurotoxin, could be effectively targeted to all classes of FcγRs on APCs and elicit enhanced antigen-reactive antibody responses [34], [35]. Thus, we think the enhanced immunity induced by E3R4 may result from targeted antigen delivery to APCs. We do not exclude other mechanisms contributing to the enhanced immune responses, such as prolonged serum half-life of Fc-fusion protein [48]. In addition, as R4 is derived from human IgG1, we speculate that administration of E3R4 which containing a modified human IgG1 Fc in humans may result in better immune responses than in mice because human Fc can bind to human FcγR on immune cells, such as macrophages and DCs, more efficiently, thus contributing to an enhanced immunity.

Previous studies have shown that the immunogenicity of HPV16 L2 aa17–36 (RG-1 epitope) is relatively low [28], [43]. In our study, we failed to detect neutralizing antibodies in the antisera from the mice immunized with E3R4 alone for four times (not shown), which may be mainly due to the weak immunogenicity of RG-1 epitope. Our results were consistent with the previous report on a chimeric adeno-associated virus-like particle bearing L2 epitopes from HPV16 and 31, which also failed to induce neutralizing antibodies when it was immunized alone [28]. A concatenated multi-type L2 fusion protein was shown to elicit more broad neutralizing antibody responses than recombinant L2 derived from a single HPV type [43], and a thioredoxin fusion protein with multiple copies of L2 peptide induced stronger immunogenicity than with single copy of L2 peptide [10]. In this study, we also generated E12R4 protein which contains 12 copies of RG-1 epitope. Unfortunately, due to the poor protein stability, E12R4 only induced lower-level of anti-HPV16 neutralizing antibodies and non-detectable cross-neutralizing antibodies against other HPV types. Consistent with our observations, Jagu et al found that HPV16 L2 aa 13–88, an unstable peptide, produced much weaker cross-neutralizing antibody responses than HPV16 L2 aa 1–88, a stable peptide containing the same amount of epitopes [43], suggesting an essential role of the stability of antigen protein in vaccine efficacy. We speculate that the vaccine potency can be further enhanced if a stable L2R4 protein containing much more L2 epitopes from multiple HPV types is developed.

It is worthy to note that unlike Freunds adjuvanted E3R4 immunization in mice, Alum-MPL adjuvanted E3R4 immunization in rabbits only induced low-titers of neutralizing antibodies against HPV16, 18 and 6. Adjuvant [24], [49], [50], [51], antigen dose, animal model and vaccine scaffold are all contributing to the final antibody responses. For example, the RG1-BPV1 VLPs (with bovine papillomavirus L1 VLP as a scaffold) formulated with Freunds adjuvant (CFA/IFA) induced much higher neutralizing antibody titers against HPV types 16, 18, 45, 58 and 5 than formulated with Alum-MPL [24]. Furthermore, even using the same adjuvant Alum-MPL, the neutralizing antibody titers against HPV types 16 and 18 were much higher in mice receiving 10 µg dose of RG1-BPV1 VLPs than those in rabbits receiving 50 µg dose (5-fold-higher dose than in mice) [24]. In our study, the adjuvant used in rabbits (Alum-MPL) is less effective than that used in mice (Freunds adjuvant), and we only used 2.35-fold-higher dose (4 nmol) in rabbits than that in mice (1.7 nmol). We also noticed that the cross-neutralization capacity of Alum-MPL adjuvanted E3R4 in rabbits in this study is lower than the Alum-MPL adjuvanted RG1-VLP, which uses papillomavirus VLP as a platform to display the RG-1epitope [25]. Papillomavirus VLPs themselves are good “adjuvant” because of their highly immunogenic surface characteristics [52], [53]. Moreover, theoretically, there are up to 360 copies of RG-1 epitope are surface exposed per chimeric RG1-VLP, while only 6 copies of RG-1 epitope are surface exposed per E3R4 protein. Thus, we reason that the differential broadness is likely resulted from the usage of different scaffolds to display the RG-1 epitope.

Taken together, our data demonstrate that a modified human IgG1 Fc can be used as a scaffold to display L2 antigen to induce cross-neutralizing antibodies and protective immunity against divergent human papillomavirus types. This type of fusion protein can be expressed with high yield and easily purified with high purity. Therefore, delivery of L2 antigen by a modified Fc scaffold opens a new avenue for pan-HPV vaccine development.
